# Effects of dietary *Clostridium butyricum* and fructooligosaccharides, alone or in combination, on performance, egg quality, amino acid digestibility, jejunal morphology, immune function, and antioxidant capacity of laying hens

**DOI:** 10.3389/fmicb.2023.1125897

**Published:** 2023-02-22

**Authors:** Uchechukwu Edna Obianwuna, Kai Qiu, Jing Wang, Hai-jun Zhang, Guang-hai Qi, Ling-ling Huang, Shu-geng Wu

**Affiliations:** ^1^Key Laboratory of Feed Biotechnology of Ministry of Agriculture and Rural Affairs, Laboratory of Quality and Safety Risk Assessment for Animal Products on Feed Hazards (Beijing) of Ministry of Agriculture and Rural Affairs, Institute of Feed Research, Chinese Academy of Agricultural Sciences, Beijing, China; ^2^Wilmar (Shanghai) Biotechnology Research & Development Center Co., Ltd, Shanghai, China

**Keywords:** *Clostridium butyricum*, laying performance, fructooligosaccharides, synbiotics, egg quality

## Abstract

The present study was conducted to evaluate the effects of *Clostridium butyricum* (CB) and fructooligosaccharide (FOS) singly or combined, on performance, egg quality, amino acid digestibility, jejunal morphology, immune function and antioxidant capacity in peak-phase laying hens. A total of 288 Hy-Line Brown laying hens (30 weeks of age) were randomly assigned to 4 dietary groups that included basal diet, basal diet +0.02% of CB (zlc-17: 1 × 10^9^ CFU/g) (PRO), basal diet +0.6% FOS (PRE), and basal diet +0.02% CB + 0.6% FOS (SYN) for 12 weeks. Each treatment had 6 replicates with 12 birds each. The results demonstrated that probiotics (PRO), prebiotics (PRE) and synbiotics (SYN) (*p* ≤ 0.05), respectively, exerted a positive effect on the performance and physiological response of the birds. There were significant increases in egg production rate, egg weight, egg mass, daily feed intake and reduced number of damaged eggs. and zero mortality rate due to dietary PRO, PRE and SYN (*p* ≤ 0.05) respectively. Also, feed conversion was improved by PRO (*p* ≤ 0.05). In addition, egg quality assessment showed that; eggshell quality was increased by PRO (*p* ≤ 0.05) and albumen indices (Haugh unit, thick albumen content, and albumen height) were enhanced by PRO, PRE and SYN (*p* ≤ 0.05). Further analysis showed that PRO, PRE and SYN (*p* ≤ 0.05), reduced heterophil to lymphocyte ratio, increased antioxidant enzymes and immunoglobulin concentration. Although spleen index was higher for PRO (*p* ≤ 0.05) group. The significant increase in villi height, villi width, villi height to crypt depth ratio and reduced crypt depth were obvious for PRO, PRE, and SYN (*p* ≤ 0.05). Furthermore, improved nutrient absorption and retention evidenced by increased digestibility of crude protein and amino acids, were notable for PRO, PRE, and SYN (*p* ≤ 0.05) group. Collectively, our findings revealed that dietary CB and FOS alone, or combined, enhanced productive performance, egg quality, amino acid digestibility, jejunal morphology, and physiological response in peak-phase laying hens. Our results would provide direction on nutritional strategies for gut enhancers and better physiological response of peak laying hens.

## Introduction

Poultry products (meat and eggs) constitutes one of the major sources of animal protein for humans. The demand for these products is on the rise due to ever increasing population and farmers must meet up with this demand, hence poultry production has increased significantly over the years. In a bid to meet up with this enormous task, feed additives such as synthetic antibiotics are commonly used as animal growth-promoters and an antimicrobial agent to enhance production and gut health. Nevertheless, synthetic antibiotics cannot be used in laying hen industry due to related safety risks with eggs (Xiang et al., 2019), and other negative effects such as drug-resistant bacteria, drug residue in animal tissue and alteration of microbiota balance, which poses a threat to both animal and consumers health. In this dimension, beneficial microorganisms such as prebiotics, probiotics and synbiotics may hold considerable promise as safe feed additives and could be harnessed by poultry industry (Adhikari et al., 2018; Salah et al., 2018; Ding et al., 2019; Macit et al., 2021).

Probiotics (PRO) are considered as live organisms or microbial feed supplements targeted at modulating the gastrointestinal tract by enhancing intestinal microbial balance (Xu et al., 2022). Probiotics have been shown to enhance laying performance (Xiang et al., 2019; Wang et al., 2021; Pan et al., 2022) and egg quality (Zhan et al., 2019; Wang et al., 2021). The additive influence of probiotics on poultry birds including the capacity of probiotics; to enhance gut development and integrity, improve and suppress immune and inflammatory response respectively, enhance feed intake and nutrient utilization by increasing activity of digestive enzymes and intestine health (Mikulski et al., 2020; Souza et al., 2021; Pan et al., 2022) have been documented. Probiotics commonly used as in-feed additives in poultry nutrition include; colonizing species of *Streptococcus*, *Lactobacillus, Clostridium*, *Bacillus*, and *Enterococcus*. *Clostridium butyricum* (CB) is a gram-positive anaerobe, mainly present in in the intestinal tract of humans and animals (Yang et al., 2010). The suitability of CB as feed additive in poultry diets are attributable to its spore-forming capacity and production of short chain fatty acids (SCFAs) such as butyric acid (Liu et al., 2018), survivability in low pH environment (Kong et al., 2011), stimulation of digestive enzymes (Duan et al., 2018) and enhancing activity of nutrient transporters (Wang et al., 2020). Myriad of beneficial effects of CB on chickens have been reported; favor growth of beneficial microbes and suppress pathogen colonization (Yang et al., 2012; Zhang et al., 2014), enhance activity of antioxidant enzymes (Liao et al., 2015; Zhan et al., 2019), immune function (Zhan et al., 2019), intestinal morphology, and oviduct health (Wang et al., 2021). These in turn promote nutrient absorption and physiological response for improved laying performance (Xiang et al., 2019; Wang et al., 2020, 2021) and albumen quality (Xiang et al., 2019; Obianwuna et al., 2022b). Nevertheless, colonization of probiotics in the gut is reliant on many factors, including nutritional status of the host, health, age, genetics, intestinal pH and availability of fermentation substrate (prebiotics). Hence, prebiotics are crucial to the survival potentials of probiotics in the gut.

Prebiotics (PRE) are referred to as indigestible foods or feed ingredients that beneficially affect the host *via* selective stimulation of growth of one or few numbers of bacteria in the colon (Mookiah et al., 2014). There are evidences in literatures that utilization of prebiotics as feed additives in poultry diets could enhance laying performance (Guo et al., 2020; Xu et al., 2020; Obianwuna et al., 2022a), egg quality (Li et al., 2017; Guo et al., 2020), and physiological response (Adhikari et al., 2018; Xu et al., 2020). Prebiotics are known to modulate the intestinal microflora *via* increase in probiotic bacteria such as *Lactobacillus* and *Bifidobacterium* (Alavi et al., 2012), inhibit colonization of gut by pathogenic bacteria *via* competitive adhesion to their binding sites on the intestinal mucosa (Adhikari et al., 2018). Thus, the prebiotic effect enhance proliferation of healthy microecological balance in the gut. Common prebiotics utilized in poultry diets include; lactulose, inulin, transgalactooligosaccharides, galactooligosaccharides, and fructooligosaccharide (FOS). FOS are non-digestible oligosaccharides due to presence of β-linkages: β-2,1 fructosyl-fructose and β-2,6 fructosyl-fructose (Zhao et al., 2013). This linkage accounts for its resistance to digestive enzymes of monogastric and selectively serves as substrate for proliferation of *Lactobacillus* spores in the intestinal tract (Ricke, 2015). Fermentation of FOS in the gut is associated with production of acetic acid and lactate acid (Rossi et al., 2005; Yang et al., 2008), which accounts for array of effects; reduction of pathogen colonization in the gut (Xu et al., 2003; Li et al., 2008) and ovary (Donalson et al., 2008; Adhikari et al., 2018), improved immune response in the host (Xu et al., 2003), enhanced villi morphometrics in the jejunum and increased enzyme activity for better nutrient absorption (Xu et al., 2003; Obianwuna et al., 2022a). This health promoting effects enhance performance and physiological status of the birds. In addition, the interaction between prebiotics and probiotics may enhance; the probiotics adaptation to the substrate (prebiotics), probiotics multiplication and invariably its synergetic effect (Hamasalim, 2016).

Combined form of probiotics and prebiotics as an in-feed additive is often considered as a synbiotics (SYN; Hassanpour et al., 2013). Synbiotic products have the capacity to enhance proliferation of existing beneficial strains in the gut and as well sustain the survival and growth of new probiotic strains (Likotrafiti et al., 2016). Previous reports showed that dietary synbiotics (FOS (prebiotics) + *Lactobacillus.plantarum* 15–1 (probiotic)) enhanced the intestinal health and immune response of broilers challenged with *E. coli* O78 (Ding et al., 2019). Several probiotic strains and FOS combined as a synbiotic product enhanced growth performance in broiler under *E. coli* challenge (Luoma et al., 2017). In another study, Zarei et al. (2018) reported that a synbiotic product (whey powder and probiotics) supplemented in broilers diets improved intestinal microflora and intestinal morphology. In addition, combination of bacillus and inulin (Abdelqader et al., 2013), probiotic and isomaltooligosaccharides (Tang et al., 2017) enhanced egg production and beneficial microbial composition in laying hens. *In ovo* feeding of synbiotics (whey powder and *Pediococcus acidilactici*) enhanced caecal microbial diversity and upregulated pathways crucial to increased egg production and egg quality (Pineda-Quiroga et al., 2019). However, Mohebbifar et al. (2013) reported no significant effect of probiotics or prebiotics on performance, egg quality and blood parameters of laying hens. In one study, Youssef et al. (2013) revealed that commercial synbiotics product improved egg production, egg mass but had no effect on albumen quality.

Evidently, in-feed application of feed additives such as probiotics, prebiotics alone or in combination (synbiotics) has the potential to modulate the gut environment and physiological response, which beneficially influence growth performance, health of the animals and product quality. Our preliminary research suggested that inclusion of FOSFOS at 0.6% and CB at 0.02% level had no adverse effect on the laying hens. Previous literatures have also shown that CB can be supplemented in the diet of laying hens at various levels; 0.5 g/kg (Xiang et al., 2019), 0.9 g/kg (Wang et al., 2020), without negative effects. Also, FOS have been included at 0.4% (Xu et al., 2003) levels in broilers diets, 0.5–1.0% (Adhikari et al., 2018), 200 mg/kg (Li et al., 2017), and 0.2–0.6% (Feng and Xia, 2019) in diet of breeder hens. To date, there are few studies which have examined effects of combined probiotic and prebiotics as a single product (Synbiotic) on laying hens and to the best of our knowledge our present study is the first to examine a combined effect of FOS and CB as a synbiotic. Therefore, the present study investigated the effects of probiotics (CB), or prebiotics (FOS) and a combination of the two (CB + FOS) on laying performance, egg quality, amino acid digestibility, intestinal morphology, antioxidant and immune function in peak-phase laying hens.

## Materials and methods

### Ethics statement

All the experimental procedures adopted in this study were in accordance to that approved by the Institutional Animal Care and Use Committee of the Feed Research Institute of the Chinese Academy of Agricultural Sciences, Beijing, China (CAAS.No20200507).

### Experimental design

Hy-Line Brown laying hens at the peak-laying phase (30-week old, laying rate = 89.0 ± 1.5%, and *n* = 288) with laying rate of similar range were randomly allocated to one of four treatment groups with 6 replicates each (n-72). The experiment lasted for 13 weeks; a 12-week test period and a 1-week feed transition period. The controlled environment of the pen house was maintained daily on: Temperature (24°C), humidity (50–80%), and 16 h of light. The laying hens were offered fresh feed (mash form) and water *ad lib* on a daily basis while being managed based on Hy-line International Online Management Guide (Hy-Line W-98 Commercial Management Guide, 2004–2006). Throughout the feeding trial, the health of the birds was stable and no medications were administered except for routine vaccination.

A 2 × 2 factorial arrangement with two levels of *C. butyricum* (0 and 0.02%) and two levels of FOS (0 and 0.6%) was designed in the present study. The dietary treatments consist of basal diet without the feed additives, basal diet+0.02% of *C. butyricum* (zlc-17: 1 × 10^9^ CFU/g) (PRO), basal diet +0.6% FOS (FOS, 99% purity) (PRE), and basal diet +0.02% *C. butyricum* + 0.6% FOS (SYN). Before the commencement of the feeding experiment, the birds were offered laying hens’ diet which is corn-soybean meal-based mash feed. The nutrient composition and nutrient level of the diet are presented in Table 1. The basal diet nutrient composition and levels are consistent with the standard proposed by National Research and Council (1994). The feed additives (*C. butyricum* and FOS) were provided by COFCO Nutrition and Health Research Institute (Beijing, China).

**Table 1:** The composition and nutrient levels of the basal and supplemented diet.

Ingredients (kg)	Control	PRO	PRE	SYN
Corn	63.4	63.40	62.15	62.15
Soybean meal	25.46	25.46	25.71	25.71
Oil	0.00	0.00	0.40	0.40
Limestone	8.76	8.76	8.76	8.76
DL-methionine	0.18	0.18	0.18	0.18
Dicalcium Phosphate	1.6	1.6	1.6	1.6
Salt	0.16	0.16	0.16	0.16
Premix (Choline Chloride)	0.25	0.25	0.25	0.25
FOS	0.0	0.00	0.6	0.6
*Clostridium butyricum*	0.00	0.02	0.00	0.02
Sodium sulfate	0.17	0.17	0.17	0.17
Phytase	0.02	0.02	0.02	0.02
Total	100	100	100	100
Nutrient Content %				
Crude protein	16.50	16.50	16.50	16.50
Calcium	3.50	3.50	3.50	3.50
Total Phosphorus	0.60	0.60	0.60	0.60
Available Phosphorus	0.39	0.39	0.39	0.39
Metabolizable Energy (MJ/kg)	11.23	11.23	11.23	11.23
SID Methionine	0.434	0.434	0.433	0.433
SID Lysine	0.796	0.796	0.798	0.798
SID Tryptophan	0.176	0.176	0.176	0.176
SID Threonine	0.560	0.560	0.561	0.561
SID methionine+ cysteine	0.653	0.652	0.651	0.651
SID Isoleucine	0.666	0.666	0.667	0.667
SID Cysteine	0.240	0.239	0.239	0.239
SID Valine	0.746	0.746	0.746	0.746
SID Arginine	1.030	1.031	1.033	1.033
SID Leucine	1.414	1.412	1.410	1.410
SID Serine	0.776	0.776	0.776	0.776
SID Glycine	0.616	0.616	0.616	0.616

The values are calculated values. AME: apparent metabolizable energy. Two Vitamin and mineral premix provided the following per kg of diets: VA: 12,500 IU; VD3: 4125 IU; VE: 15 IU; VK: 2 mg; VB1: 1 mg; VB2: 8.5 mg; VB6: 8 mg; VB12: 5 mg; calcium pantothenate: 50 mg; niacin: 32.5 mg; biotin: 2 mg; folic acid: 5 mg; choline: 500 mg; Mn: 65 mg; I: 1 mg; Fe: 60 mg; Cu: 8 mg; Zn: 66 mg; Se: 0.3 mg.

Control, basal diet; PRO, basal diet + 0.02% *Clostridium butyricum*; PRE, basal diet + 0.6%; FOS, fructooligosaccharides; SYN, basal diet + 0.02% *Clostridium butyricum* + 0.6% FOS.

### Measurement of laying performance parameters

During the experiment, egg production rate, egg weight, damaged eggs (shells, misshapen and cracked) and mortality rate were recorded daily on replicate basis. Feed consumption was recorded every fortnight on replicate basis. The data collected was used to calculate the following variables; egg mass, average egg weight (AEW), hen-day production (HDP), average daily feed intake (ADFI), and feed conversion ratio (FCR) for the whole feeding period. The HDP and the ADFI were adjusted for mortality.

### Egg quality assessment

On the last day of each of the 4-week period (week 4, 8, and 12), respectively, three eggs were collected from each replicate with the weight close to replicate range (18 eggs per treatment group). The collected eggs were placed under room temperature and assessment of egg quality was done within 24 h. Each egg was weighed, recorded, then broken and the albumen and yolk are separated with the egg separator. The individual components were then weighed, respectively. The eggshells were cleaned of albumen remains and air-dried for 48 h, then the eggshell weight was obtained. The relative proportion of albumen, yolk and shell was obtained as their weight/egg weight × 100% (Sarlak et al., 2021). Eggshell thickness (average of 3 points (air cell, equator, and sharp end) around the eggshell) (Mwaniki et al., 2018) and egg shell breaking strength were, respectively, determined with Eggshell Thickness Gauge and Egg Force Reader (ESTG-1, ORKA Technology Ltd., Ramat HaSharon, Israel). Albumen height, Haugh Unit and yolk color measurements were detected with an automatic Egg Analyzer (ORKA Food Technology Ltd., Ramat HaSharon, Israel). Further analysis of egg quality; the weighed albumen was separated further by moving it through using a 60-mesh sieve and allow it to stand for 30 s. the thick portion remained glued on the sieve and was weighed as thick albumen while the filtrate was weighed as thin albumen (Zhou et al., 2021).

### Determination of serum and blood biochemical indices

At the end of the study period (12th week), 24 birds [six birds from each group (1 per replicate)] were selected for sample collection after 12 h fasting. About 5 ml of blood were collected from the jugular veins, then kept in a micro-anticoagulant tube in slanting position for 30 min, centrifuged at 1,500 x g for 15 min (Tang et al., 2017). The harvested plasma was transferred to Eppendorf tubes (1.5 ml) and stored at –20°C until analysis. The whole blood samples were put together in an ice pack and transported to the laboratory for hematology analysis within 1 h of collection. For hematological indices analysis, an automated hematology analyzer (Model: BC-2800 Vet, Mindray, Shenzhen, China) was used. Red blood cell indices, MCH, MCV and mean cell hemoglobin concentration (MCHC), were calculated (Jain, 1993).

Before analysis of biochemical indices, the serum was thawed at 4°C and kept at low temperature during the whole process in order to avoid activation of enzymes. Determination of glutathione peroxidase (GST), glutathione peroxidase (GSH-Px), total antioxidant capacity (T-AOC), total superoxide dismutase (T-SOD), catalase (CAT), and malondialdehyde (MDA) were achieved using an assay kits from ML Bio and Jiancheng Bioengineering Institute (Nanjing, China), and measured spectrophotometrically (Shimadzu, model UV-1800, Tokyo, Japan). Serum concentrations of immunoglobulins A (IgA), immunoglobulins G (IgG), immunoglobulins M (IgM) and complement proteins; C3 and C4 were determined with the corresponding chicken ELISA kits. All standards were tested in duplicate and concentrations of IgA, IgG, IgM, C3, and C4 were determined using standard curves constructed from the standards run on the plate. All the ELISA kits adopted in the study are of high specificity and sensitivity for chickens, and all procedures were done according to the manufacturer’s instructions.

### Intestine sample collection and jejunal morphology analysis

At the end of the study (12th week), 24 birds [six birds from each group (1 per replicate)] were selected for sample collection after 12 h fasting. The selected birds were euthanized with pentobarbital sodium (100 mg/kg BW) intravenously and cut open under aseptic conditions. The heart, liver, magnum and spleen of each bird were weighed and the organ index was calculated weight of organ (g)/body weight (g) × 100%. The jejunal samples of each bird were processed following that established in Gungor and Erener (2020). About three-centimeter length of tissue from jejunum were flushed in physiological saline solution and placed in 10% buffered formalin, kept at 4°C for analysis.

The fixed jejunal samples were processed and stained with hematoxylin–eosin, while adopting the standard techniques for histology examination (Yamauchi et al., 2010). Images were observed with the aid of a microscope (An Olympus BX43 microscope; Olympus Corp., Tokyo, Japan). The villi morphometrics were obtained; ten intact villi of each sample and corresponding crypts were selected, measured, and the average value was obtained. The measurements were made with a software (Caseviewer Image). The measurements include; villi height (VH: measured from the top of the villus to the villus-crypt junction), the villus width (VW: was measured at the middle point of the villus), crypt depth (CD: from the base up to the crypt–villus transition region), the VH-CD ratio (measured as VH/CD), villi surface area (VSA: π × VW × VH) (Wang et al., 2016; Thiam et al., 2021).

### Apparent fecal amino acid digestibility

At the end of 12 weeks, 3 birds were chosen from each replicate and put in a metabolic cage with collection tray for fecal samples. The fecal samples were collected on replicate basis at 12 h intervals daily for 3 days. Contamination of fecal samples were avoided by ensuring that feed, feathers and other foreign materials were completely excluded from fecal samples during collection. The fecal samples were then weighed, pooled together for each treatment group, and kept in sealed bags at –20°C (Zhou et al., 2021). Prior to amino acid analysis, the fecal samples were thawed and oven-dried at 65°C for 72 h, then weighed again and pulverized into finely ground powder that can pass through a 0.05 mm mesh. Amino acids determinations from samples of feed and feces was performed by HPLC, following an established method by Varzaru et al. (2013). The HPLC system was Finnigan Surveyor Plus and HyperSil BDS C18 column, size 250 × 4.6 mm, 5 μm (Thermo-Electron Corporation, Waltham, MA). For apparent fecal amino acid digestibility analysis, the feed intake and the fecal weight (dry matter basis) from each metabolic cage were calculated. The apparent fecal amino acid digestibility was calculated as: Apparent Amino acid Digestibility = 1 ˗ (amino acid concentration in the feces ×feces weight)/(amino acid concentration in the feed × feed intake) × 100%.

### Statistical analysis

The experiment consists of four groups with 6 replications each, in a completely randomized design to ensure random allocation of birds to treatments. All the data generated in this study were subjected to using one-way analysis of variance (ANOVA), this ensures that there is no biasness in respect to data normality and equality of variance assumptions (Nwachukwu et al., 2021). Replicates were used as experimental units, the data were presented as mean and pooled standard error of mean (SEM), while the level of significance was considered at *p* ≤ 0.05. Overall differences between the dietary treatments were analyzed using one-way analysis of variance (ANOVA), and the general linear model procedure. The statistical package used was SPSS software, version 17.0 (SPSS Inc., Chicago, II, United States).

## Results

We conducted two trials at the same time. This design can save trial resources and did not affect the research objectives of the two trials. Although the data from two groups are shared, the data analysis is completely different, and one part of the trial have been published in Obianwuna et al. (2022b).

### Laying performance

The effects of PRO, PRE and SYN on egg production and performance of laying hens are presented in Table 2. The results of this study demonstrated that at the end of week 4, average egg weight, egg mass, hen-day production, feed intake, feed conversion ratio and number of damaged eggs were not influenced by each of the PRO, PRE, and SYN (*p* > 0.05) group, while mortality rate was influenced by PRO, PRE and SYN (*p* ≤ 0.05) respectively. At the end of week 8; average egg weight and mortality rate were influenced by the PRO, PRE and SYN (*p* ≤ 0.05), egg mass and hen-day production were increased by PRO and SYN (*p* ≤ 0.05) while the effect of PRE (*p* > 0.05) was comparable to control, feed conversion ratio was enhanced by the PRO (*p* ≤ 0.05) but no diet effect was observed for PRE and SYN (*p* > 0.05) group, and no treatment effect (*p* > 0.05) was observed for feed intake and number of damaged eggs. At the end of week 12; average egg weight was higher for PRO (*p* ≤ 0.05) group but the effects of PRE and SYN (*p* > 0.05) were not significant, egg mass, hen-day production, mortality rate and feed intake were improved by PRO, PRE and SYN (*p* ≤ 0.05), number of damaged eggs and feed intake were not influenced by the dietary treatments (*p* > 0.05). The results for the overall period (weeks 1–12) showed that; average egg weight, and feed conversion ratio were not influenced by neither PRO, PRE nor SYN (*p* > 0.05), egg mass, feed intake, mortality rate and number of damaged eggs were influenced by PRO, PRE and SYN (*p* ≤ 0.05), and hen-day production was increased by PRO and PRE (*p* ≤ 0.05) but the effect of SYN (*p* > 0.05) was comparable to the control.

**Table 2:** Effects of dietary treatments on the performance of laying hens.

Items	Control	PRO	PRE	SYN	SEM	*p*-value
Week 1 ~ 4						
AEW (g)	61.41	61.40	60.89	58.80	0.90	0.44
Egg mass (g)	58.30	59.71	59.20	57.00	0.95	0.41
HDP %	95.12	97.17	97.22	92.91	1.12	0.38
Number of Damaged eggs	7.83	3	4.83	5.66	1.03	0.31
Mortality %	5.55	0	0	0	0.00	0.02
ADFI (g)	116.58	120.51	119.18	118.98	3.22	0.42
FCR	2.00	2.02	2.01	2.11	0.06	0.57
Week 5 ~ 8						
AEW (g)	61.84^b^	63.85^a^	62.45^a^	61.24^a^	0.64	0.01
Egg mass (g)	57.65^c^	61.98^a^	59.30^bc^	60.54^ab^	1.12	0.01
HDP %	93.34^b^	97.18^a^	94.89^ab^	96.91^a^	2.53	0.02
Number of Damaged eggs	8.83	4.83	4.5	5.5	0.80	0.20
Mortality %	8.89	0	0	0	0.01	< 0.01
Feed intake (g)	112.82	114.35	113.74	115.23	3.47	0.75
FCR	1.97^a^	1.84^b^	1.92^ab^	1.90^ab^	0.06	0.05
Week 9 ~ 12						
AEG (g)	59.98^b^	62.04^a^	60.51^b^	60.85^ab^	0.94	0.02
Egg mass (g)	53.85^b^	58.45^a^	58.16^a^	58.33^a^	1.44	0.01
HDP %	89.76^b^	94.30^a^	96.13^a^	95.86^a^	1.97	0.01
Number of damaged eggs	6.17	3.5	4	4.67	1.10	0.79
Mortality %	1.67	0	0	0	0.00	0.41
ADFI (g)	121.17^b^	129.68^a^	132.41^a^	132.65^a^	3.21	0.01
FCR	2.25	2.22	2.28	2.28	0.09	0.72
Week 1 ~ 12						
AEG (g)	61.06	62.43	60.95	60.63	0.69	0.07
Egg mass (g)	56.60^b^	60.08^a^	58.89^a^	58.62^a^	0.96	0.01
HDP %	92.70^b^	96.23^a^	96.08^a^	95.23^ab^	1.27	0.03
Number of damaged eggs	7.61^a^	3.77^c^	4.44^b^	5.27^b^	0.08	0.01
Mortality %	5.37	0.00	0.00	0.00	0.01	< 0.01
ADFI (g)	116.87^b^	121.51^a^	121.78^a^	122.29^a^	2.05	0.04
FCR	2.07	2.02	2.07	2.09	0.04	0.32

Control-basal diet, PRO-basal diet + 0.02% *Clostridium butyricum* (CB), PRE-basal diet + 0.6% fructooligosaccharides (FOS), SYN-basal diet + 0.02% *Clostridium butyricum* + 0.6% FOS. SEM. Standard error of mean.

AEW, average egg weight; HDP, Hen day production; ADFI, average daily feed intake; FCR, Feed conversion ratio.

Data represent mean of six replicates of three hen each. Means within a row with different superscripts a, b, c differs significantly (*p* ≤ 0.05), while same superscripts are not significantly different. *p* values (*p* > 0.05) are not statistically different.

### Egg quality assessment

The effects of PRO, PRE and SYN on egg quality of laying hens are presented in Table 3. At the end of week 4; the relative weight of albumen and yolk were influenced by PRE and SYN (*p* ≤ 0.05) while no significant effect was observed for PRO (*p* > 0.05), eggshell thickness was enhanced by PRO (*p* ≤ 0.05) but the effects of PRE and SYN (*p* > 0.05) were not significant. The eggshell strength, yolk color and albumen indices (albumen height, Haugh unit, and thick to thin albumen ratio) were not influenced by PRE, PRO nor SYN (*p* > 0.05). At the end of week 8; eggshell thickness, yolk color and eggshell weight were not influenced by any of PRO, PRE, and SYN (*p* > 0.05), relative weight of albumen was enhanced by SYN (*p* ≤ 0.05) while the effects of PRO and PRE (*p* > 0.05) were not significant, relative yolk weight was influenced by PRO and SYN (*p* ≤ 0.05) but not PRE (*p* > 0.05), eggshell strength was enhanced by PRO (*p* ≤ 0.05) but the effects of PRE and SYN (*p* > 0.05) were comparable to control, albumen height and Haugh unit were significantly increased by PRO, PRE and SYN (*p* ≤ 0.05) respectively, and thick to thin albumen ratio was enhanced by PRO and SYN (*p* ≤ 0.05) but not PRE (*p* > 0.05). At the end of week 12; the relative weight of albumen and yolk, yolk color, and albumen indices (albumen height, Haugh unit and thick to thin albumen ratio), were significantly improved by PRO, PRE and SYN, respectively. Also, eggshell thickness, eggshell strength, and eggshell weight were not influenced by any of PRO, PRE, and SYN (*p* > 0.05). The effect of SYN on albumen indices were numerically higher compared to PRO and PRE, at the end of week 8 and 12.

**Table 3:** Effects of dietary treatments on the egg quality of laying hens.

Items	Control	PRO	PRE	SYN	SEM	*p*-value
Week 4						
Relative Albumen weight %	59.58^c^	61.60^bc^	62.70^ab^	63.62^a^	1.33	0.02
Relative yolk weight %	29.50^a^	27.80^ab^	26.90^bc^	25.79^c^	0.88	0.03
Relative shell weight %	10.92	10.60	10.40	10.59	0.32	0.14
Shell thickness (mm)	4.69^b^	4.86^a^	4.57^b^	4.65^ab^	0.09	0.01
Shell strength (N)	45.40	46.28	46.94	47.88	3.81	0.54
Yolk color	7.11	7.17	6.61	6.83	0.44	0.96
Albumen height (mm)	6.81	7.12	6.77	6.86	0.68	0.87
Haugh units	80.23	81.09	78.83	81.22	0.73	0.78
Thick to thin albumen ratio	1.00	1.09	1.13	1.20	0.12	0.29
Week 8						
Relative albumen weight %	60.15^b^	61.43^ab^	60.59^b^	62.41^a^	0.99	0.01
Relative yolk weight %	29.01^a^	26.67^c^	28.81^ab^	27.14^bc^	0.86	0.01
Relative shell weight %	10.84	11.90	10.60	10.35	0.44	0.17
Shell thickness (mm)	4.49	4.74	4.70	4.76	0.17	0.08
Shell strength (N)	36.20^b^	44.27^a^	41.00^ab^	40.61^ab^	3.76	0.03
Yolk color	5.72	8.50	6.11	6.17	2.21	0.55
Albumen height (mm)	7.80^c^	8.93^b^	8.66^b^	9.72^a^	0.47	0.01
Haugh units	86.95^b^	93.27^a^	91.84^a^	96.23^a^	2.84	0.03
Thick to thin albumen ratio	1.08^b^	1.87^a^	1.24^b^	1.95^a^	0.27	0.01
Week 12						
Relative albumen weight %	58.24^b^	63.78^a^	62.71^a^	63.94^a^	1.22	0.01
Relative yolk weight %	31.38^a^	25.86^bc^	26.95^b^	25.44^c^	1.00	0.01
Relative shell weight %	10.22	10.36	10.34	10.62	0.47	0.28
Shell thickness (mm)	4.43	4.59	4.61	4.54	0.18	0.54
Shell strength (N)	36.97	39.05	38.25	39.44	2.53	0.48
Yolk color	5.72^b^	7.17^a^	6.72^a^	7.00^a^	0.40	0.01
Albumen height (mm)	6.19^c^	7.90^b^	8.58^a^	8.65^a^	0.34	0.01
Haugh units	76.16^d^	87.36^c^	91.59^b^	95.16^a^	2.00	0.01
Thick to thin albumen ratio	1.14^c^	1.52^b^	1.59^ab^	1.75^a^	0.12	0.01

Control-basal diet, PRO-basal diet + 0.02% *Clostridium butyricum* (CB), PRE-basal diet + 0.6% fructooligosaccharides (FOS), SYN-basal diet + 0.02% CB + 0.6% FOS.SEM-standard error of mean.

Data represent mean of six replicates of three hen each. Means within a row with different superscripts a, b, c differs significantly (*p* ≤ 0.05), while same superscripts are not significantly different. *p* values (*p* > 0.05) are not statistically different.

### Hematological and serum biochemical indices

The effects of PRO, PRE and SYN on the hematological indices of laying hens are presented in Table 4. Hematological indices including WBC and H/L ratio were significantly influenced by PRO, PRE and SYN (*p* ≤ 0.05) respectively. The H/L values for PRO was significantly lower (*p* ≤ 0.05), compared to that of PRE and SYN. The effect of PRO (*p* ≤ 0.05) on MCV and monocytes, and SYN (*p* ≤ 0.05) on heterophil were significant but not PRE (*p* > 0.05) effect. Other indices including; RBC, Hb, PCV, MCH, MCHC, platelets, lymphocytes, basophil and eosinophil were not influenced by any of the PRO, PRE and SYN (*p* > 0.05).

**Table 4:** Effects of dietary treatments on the Hematological indices of laying hens.

Items	Control	PRO	PRE	SYN	SEM	*p*-value
WBC (× 10^9^/L)	12.13^c^	17.23^ab^	16.54^b^	19.20^a^	1.52	< 0.01
RBC (× 10^12^/L)	2.26	2.30	2.42	2.24	0.11	0.06
Hb (g/L)	71.00	73.80	74.67	69.80	4.89	0.83
PCV (%)	35.03	35.00	37.65	35.30	1.64	0.99
MCV (fL)	155.10^ab^	151.90^b^	155.70^ab^	157.00^a^	3.63	0.04
MCH (Pg)	31.43	30.28	30.92	31.10	1.05	0.55
MCHC (g/L)	202.67	199.40	198.30	198.00	4.50	0.16
Platelets (× 10^9^/L)	11.67	8.40	12.00	11.20	1.90	0.08
Heterophil (× 10^9^/L)	6.25^b^	7.63^b^	8.55^ab^	10.88^a^	1.81	0.05
Lymphocytes (× 10^9^/L)	5.19	7.85	4.63	6.00	1.47	0.08
H/L	1.20^b^	0.99^c^	1.42^a^	1.36^a^	0.18	0.03
Monocyte (× 10^9^/L)	0.15^a^	0.59^b^	0.80^ab^	1.04^a^	0.27	0.02
Eosinophil (× 10^9^/L)	0.05	0.17	0.27	0.08	0.24	0.33
Basophil (× 10^9^/L)	0.98	0.95	2.25	1.33	0.83	0.09

Control-basal diet, PRO-basal diet + 0.02% *Clostridium butyricum* (CB), PRE-basal diet + 0.6% fructooligosaccharides (FOS), SYN-basal diet + 0.02% CB + 0.6% FOS. SEM, standard error of mean.

WBC, White blood cells count; RBC, Red blood cells count; Hb, Hemoglobin count; PCV, Packed cell volume; MCV, Mean corpuscular volume; MCH, Mean corpuscular haemoglobin; MCHC, Mean corpuscular hemoglobin concentration; H/L, heterophil/lymphocyte.

Data represent mean of six replicates of three hen each. Means within a row with different superscripts a, b, c differs significantly (*p* ≤ 0.05), while same superscripts are not significantly different. *p* values (*p* > 0.05) are not statistically different.

The effects of PRO, PRE and SYN on serum biochemical indices of laying hens are shown in Table 5. The serum biochemical indices were significantly influenced by dietary PRO, PRE and SYN (*p* ≤ 0.05) respectively, as evidenced by increased antioxidant enzymes (T-SOD, CAT, GSH-Px and GST) and reduced level of MDA, compared to the control group. Antioxidant enzymes; T-SOD, CAT, GSH-Px and GST, were numerically higher in the SYN group compared to other dietary treatments. Among dietary treatments, there were no significant differences in T-SOD, GSH-Px and GST (*p* > 0.05), while no influence of neither PRE, PRO nor SYN was notable for T-AOC (*p* > 0.05). The concentration of IgA was enhanced by each of the PRO, PRE and SYN (*p* ≤ 0.05) treatments, IgM was increased by PRE and SYN (*p* ≤ 0.05) but not by PRO (*p* > 0.05), IgG and complement protein C3 was increased by SYN (*p* ≤ 0.05) but no treatment effect was notable for PRE and PRO (*p* > 0.05). The complement protein C4 was not influenced by any of the PRO, PRE and SYN (*p* > 0.05).

**Table 5:** Effects of dietary treatments on the serum antioxidant and immune capacity of laying hens.

Items	Control	PRO	PRE	SYN	SEM	*p*-value
MDA (nmol/mL)	8.54^a^	5.07^b^	2.56^c^	2.52^c^	0.91	< 0.01
CAT (U/mL)	9.67^d^	13.34^c^	15.29^b^	15.88^a^	0.28	< 0.01
T-SOD (U/mL)	107.41^b^	143.81^a^	148.73^a^	148.61^a^	6.92	< 0.01
T-AOC (U/mL)	11.50	13.18	14.32	15.42	1.75	0.37
GSH-Px (ng/L)	53.46^b^	76.86^a^	78.61^a^	91.20^a^	8.94	< 0.01
GST (ng/mL)	16.57^b^	18.92^a^	19.56^a^	19.30^a^	0.50	0.01
IgG (ug/mL)	58.33^b^	65.65^ab^	66.85^ab^	71.64^a^	5.04	0.01
IgM (ng/mL)	2335.50^b^	3325.00^ab^	4080.83^a^	4168.33^a^	509.51	< 0.01
IgA (ng/mL)	4762.78^b^	6104.44^a^	5918.33^a^	6146.11^a^	218.80	0.01
C3 (mg/mL)	0.073^b^	0.085^b^	0.085^b^	0.107^a^	0.007	0.01
C4 (mg/mL)	0.044	0.048	0.045	0.048	0.002	0.43

Control-basal diet, PRO-basal diet + 0.02% *Clostridium butyricum*, PRE-basal diet + 0.6% FOS-fructooligosaccharides, SYN-basal diet + 0.02% *Clostridium butyricum* + 0.6% FOS. SEM. standard error of mean.

MDA, malondialdehyde; CAT, catalase; T-SOD, total superoxide dismutase; T-AOC, total antioxidant capacity; GSH-Px, glutathione peroxidase; GST, glutathione transferase; IgG, immunoglobulin G; IgM, immunoglobulin M; IgA, immunoglobulin A; C3 and C4, complement proteins.

Data represent mean of six replicates of three hen each. Means within a row with different superscripts a, b, c differs significantly (*p* ≤ 0.05), while same superscripts are not significantly different. *p* values (*p* > 0.05) are not statistically different.

### Apparent fecal amino acid digestibility

The effects of PRO, PRE and SYN on the apparent fecal amino acid digestibility of laying hens are listed in Table 6. The digestibility of crude protein was enhanced by the PRO, PRE and SYN (*p* ≤ 0.05), respectively. Significant differences due to dietary treatments (*p* ≤ 0.05), were notable for digestibility of essential amino acids (threonine, isoleucine, leucine, phenylalanine, lysine, arginine and tryptophan) and non-essential amino acids (serine, glycine, cysteine, and tyrosine). Threonine, serine, phenylalanine, and lysine were influenced by PRO and PRE (*p* ≤ 0.05) while the effect of SYN (*p* > 0.05) was comparable to control, cysteine, isoleucine, leucine, arginine, and tyrosine were improved by PRO (*p* ≤ 0.05) but the effects of PRE and SYN (*p* > 0.05) was not notable, glycine was enhanced by PRO, PRE and SYN (*p* ≤ 0.05), respectively and tryptophan was increased by PRE and SYN (*p* ≤ 0.05) but not PRE (*p* > 0.05). There was no significant effect of PRO, PRE and SYN (*p* > 0.05) on digestibility of other amino acids.

**Table 6:** Effects of dietary treatments on apparent fecal amino acid digestibility coefficient of laying hens.

Apparent digestibility %	Control	PRO	PRE	SYN	SEM	*p*-value
Crude protein	59.20^b^	70.64^a^	71.98^a^	75.50^a^	5.37	0.05
Asparagine	75.29	81.53	81.23	80.31	4.25	0.07
Threonine	69.48^b^	76.95^a^	76.73^a^	75.35^ab^	5.41	0.01
Serine	77.63^b^	83.24^a^	83.41^a^	82.21^ab^	4.24	0.01
Glutamine	84.57	88.96	88.62	87.74	2.97	0.09
Proline	80.43	84.80	86.43	86.32	3.16	0.18
Glycine	−3.14^b^	25.75^a^	23.71^a^	22.78^a^	16.70	0.03
Alanine	65.77	73.41	71.22	71.95	5.64	0.10
Cysteine	73.58^b^	80.01^a^	76.51^ab^	77.07^ab^	4.60	0.03
Valine	73.91	79.92	77.34	78.46	4.12	0.09
Methionine	83.01	87.55	85.96	86.09	3.05	0.09
Met+cys	79.28	84.57^a^	82.34	82.64	3.62	0.09
Isoleucine	74.16^b^	81.48^a^	79.23^ab^	78.70^ab^	4.34	0.05
Leucine	80.21^b^	85.84^a^	83.43^ab^	83.31^ab^	3.13	0.03
Tyrosine	80.54^b^	88.88^a^	85.69^ab^	80.09^b^	3.60	0.02
Phenylalanine	89.25^ab^	94.66^a^	90.69^a^	82.83^b^	2.45	0.03
Histidine	51.02	63.59	52.71	40.37	8.00	0.06
Lysine	73.19^b^	80.23^a^	79.27^a^	78.06^ab^	4.52	0.01
Arginine	83.97^b^	87.84^a^	86.89^ab^	84.34^ab^	2.67	0.02
Tryptophan	73.55^c^	77.22^bc^	83.93^a^	80.75^ab^	4.63	0.01

Control-Basal diet; PRO-basal diet + 0.02% *Clostridium butyricum*, (C.B), PRE-basal diet + 0.6% Fructooligosaccharides (FOS), SYN-basal diet + 0.02% C. B+ 0.6% FOS. SEM- standard error of mean. Met + cys- methionine cysteine.

Data represent mean of six replicates of three hen each. Means within a row with different superscripts a, b, c differs significantly (*p* ≤ 0.05), while same superscripts are not significantly different. *p* values (*p* > 0.05) are not statistically different.

### Organ index and jejunal villi morphological structure

The organ weights of the magnum, spleen, heart, and liver (expressed as organ index) of laying hens offered dietary PRO, PRE and SYN are listed in Table 7. The organ index of magnum, heart, and liver were not influenced by any of the PRO, PRE and SYN (*P* > 0.05) group. The organ index of the spleen was increased by PRO (*p* ≤ 0.05) while no significant effect was reported for PRE and SYN (*P* > 0.05). The effects of PRO, PRE and SYN on the jejunal villi morphometry of laying hens are presented in Table 8. There were significant increases in VH, VSA, VH:CD and decrease in CD due to dietary effect of PRO, PRE and SYN (*p* ≤ 0.05) respectively. The villi width was increased by PRO and SYN (*p* ≤ 0.05) but not PRE (*P* > 0.05). All villi morphometrics were numerically higher in the PRO and SYN group compared to that of PRE.

**Table 7:** Effects of dietary treatments on the organ index of laying hens.

Items (%)	Control	PRO	PRE	SYN	SEM	*p*-value
Magnum	2.01	2.00	1.78	1.84	0.25	0.35
Spleen	0.09^b^	0.11^a^	0.09^b^	0.08^b^	0.01	0.04
Heart	0.36	0.37	0.35	0.36	0.04	0.79
Liver	1.38	1.65	1.66	1.5	0.2	0.15

Control-Basal diet; PRO- basal diet + 0.02% *Clostridium butyricum* (CB), PRE-basal diet + 0.6% Fructooligosaccharides (FOS), SYN-basal diet + 0.02% C. B+ 0.6% FOS. SEM, standard error of mean.

Data represent mean of six replicates of three hen each. Means within a row with different superscripts a, b, c differs significantly (*p* ≤ 0.05), while same superscripts are not significantly different. *p* values (*p* > 0.05) are not statistically different.

**Table 8:** Effects of dietary treatments on the jejunal villi morphometry of laying hens.

Items	Control	PRO	PRE	SYN	SEM	*p*-value
VH (μm)	930.70^b^	1310.60^a^	1290.00^a^	1327.86^a^	37.36	0.03
VW (μm)	139.20^c^	210.10^a^	155.00^bc^	174.56^b^	4.57	< 0.01
CD (μm)	150.01^a^	110.09^bc^	119.08^b^	99.03^c^	7.70	< 0.01
VH:CD	6.20^c^	11.90^a^	10.83^b^	13.40^a^	0.75	< 0.01
VSA (mm^2^)	0.41^c^	0.87^a^	0.63^b^	0.73^b^	0.03	0.02

Control-Basal diet; PRO- basal diet + 0.02% *Clostridium butyricum* (CB), PRE-basal diet + 0.6% Fructooligosaccharides (FOS), SYN-basal diet + 0.02% C. B+ 0.6% FOS. SEM, standard error of mean.

VH, villi height; VW, villi width; CD, crypt depth; VH, CD-villi height to crypt depth ratio; VSA, villi surface area.

Data represent mean of six replicates of three hen each. Means within a row with different superscripts a, b, c differs significantly (*p* ≤ 0.05), while same superscripts are not significantly different. *p* values (*p* > 0.05) are not statistically different.

## Discussion

To promote intestinal gut health and animal product quality in poultry industry, attempts have been made to enhance utilization of natural feed additives which are safe. Earlier reports have shown that novel feed additives such as prebiotics, probiotics or synbiotics could be used in poultry production as growth and gut enhancers (Yang et al., 2020; Song et al., 2022). Thus, the present research lends supporting evidence that supplementation of CB, FOS singly or their combination in diet of laying hens improved laying performance, egg quality, apparent fecal amino acid digestibility, blood indices and serum biochemical parameters.

### Laying performance and egg quality

In the present study, our findings revealed that probiotics, prebiotics and synbiotics enhanced laying performance, egg mass, feed intake and feed efficiency. Our findings are in line with previous reports on the beneficial effects of dietary probiotics (Darsi and Zhaghari, 2021; Souza et al., 2021; Pan et al., 2022), prebiotics (Xu et al., 2020; Zhou et al., 2021) and synbiotics (Luoma et al., 2017; Song et al., 2022) on egg production rate. There are evidences of improved laying performance due to supplementation of CB (Xiang et al., 2019;Zhan et al., 2019; Wang et al., 2021) and FOS (Li et al., 2017; Obianwuna et al., 2022a) in diets of laying hens. Further, CB (Zhan et al., 2019; Wang et al., 2021), FOS (Li et al., 2017), and synbiotics (Tang et al., 2017), have been reported to enhance egg mass. In addition, we observed an increased feed intake in the probiotics and synbiotics group while feed efficiency was significantly improved with probiotics. This is in line with other reports in laying hens, that CB enhanced feed efficiency (Xiang et al., 2019; Wang et al., 2020), while synbiotics enhanced feed intake (Tang et al., 2017). The positive effects of diets on laying performance, egg mass and feed efficiency could be attributable to availability of nutrients necessary for metabolism, maintenance and egg production (Markowiak and Śliżewska, 2018), probably because probiotics and prebiotics modulate gut structure for efficient nutrient absorption (Swiatkiewicz et al., 2015; Tang et al., 2017; Xiang et al., 2019). Also, gut fermentation effects are linked with production of short-chain fatty acids which nourish the enterocytes, protect the villi from pathogen invasion and invariably increase microvilli structures for nutrient absorption (Hajiaghapour and Rezaeipour, 2018; Liu et al., 2018). Thus, the probiotic-prebiotic fermentation effect on the gut (Yousaf et al., 2016; Xiang et al., 2019) explains the synergistic effect on laying performance. In addition, the improved crude protein digestibility of key amino acids essential for egg production may be a key factor to improved performance (Phuoc et al., 2019). Conversely, there are also reports that probiotics (Shi et al., 2020; Ray et al., 2022), CB (Wang et al., 2020), prebiotics (Li et al., 2017), FOS (Li et al., 2017), and synbiotics (Tang et al., 2017), had no influence on laying performance. Similarly, no effect on feed efficiency (Upadhaya et al., 2019) and feed intake (Abdelqader et al., 2013) due to dietary probiotics and synbiotics, respectively. The variations may be explained by the differences in viable counts of microorganisms, strains, and supplementation dosage. Further analysis showed that, zero mortality was recorded in feed additive groups, which agrees with previous reports on zero mortality due to dietary CB (Xiang et al., 2019) and prebiotics (Li et al., 2017). The enhancement effect of the feed additives on the animal health may explain the zero-mortality status. These aforementioned positive findings on laying performance and feed efficiency can be accrued to better utilization of dietary feed additives by the birds.

Egg quality is critical to consumer acceptance of table eggs and utilization by the food industry. Our findings revealed that egg shell strength was enhanced with feed additives compared to the control. This is line with previous reports that dietary CB (Xiang et al., 2019; Zhan et al., 2019) and prebiotics (Li et al., 2017; Xu et al., 2020) improved egg shell strength in laying hens. The increased eggshell strength and eggshell thickness may be due to fermentation effect in the gut which favors mineral absorption and retention with resultant effect on shell gland (Świątkiewicz et al., 2010). Contrarily, there are evidences that CB (Wang et al., 2020), FOS (Li et al., 2017), and synbiotics (Song et al., 2022) had no effect on egg shell strength. The non-significant influence on egg shell strength may be due to age of the hens and laying phase (Guo et al., 2020). Yolk color is an intrinsic trait that is of priority to consumers. In this study, we observed a significant increase in yolk color at the end of the feeding trial. In line with our findings, CB (Wang et al., 2020, 2021) and prebiotics (Guo et al., 2020) enhanced yolk color in laying hens. The enhanced yolk color may be due to potentials of the feed additives to degrade nutrients in the feed and long-term absorption may influence yolk color though the underlying principle may not be clear. Although some studies found no significant improvement in yolk color with probiotics (Lei et al., 2013) and prebiotics (Li et al., 2017). The variations in studies may be due to the composition of the feed supplement used. In addition, improved albumen quality would mean extended egg shelf life during storage and more value for its utilization by the health and food industry (Sun et al., 2016; Zhang et al., 2020). Our findings demonstrated a significant improvement in albumen quality (albumen height, Haugh unit and thick to thin albumen ratio) due to probiotics, prebiotics and the two-fold synergistic effect of synbiotics. Dietary CB have been reported to enhance albumen height (Zhan et al., 2019; Wang et al., 2020, 2021; Obianwuna et al., 2022b) and albumen crude protein (Xiang et al., 2019). Prebiotics including marine derived polysaccharides and FOS enhance albumen height and Haugh unit (Guo et al., 2020; Obianwuna et al., 2022a). Increased albumen height and Haugh unit reflects abundance of ovomucin in thick albumen fraction (Aobai et al., 2020). In the study of He et al. (2017), low protein diets could alter albumen secretion in the magnum due to reduction in albumen proteins, ovomucin inclusive. The study of Zhou et al. (2021) highlighted the critical role of high dietary crude protein and amino acid in albumen synthesis. The improvement in albumen quality may be explained by enhanced crude protein and amino acid digestibility that is crucial to albumen synthesis. This claim is supported in the previous reports that prebiotics and probiotics enhance protein digestibility and utilization (Zhang et al., 2016; Ahiwe et al., 2020). Further, the previous studies of Adhikari et al. (2018) and Wang et al. (2021) have demonstrated that FOS and CB respectively, can enhance oviduct health in laying hens. Thus, stable oviduct health may be a contributory factor to improved albumen synthesis. Conversely, CB (Xiang et al., 2019), prebiotics (Xu et al., 2020), and synbiotics (Song et al., 2022), were reported to have no effect on albumen height and Haugh unit. Variations may be due to different compounds that make up the feed additives and the supplementation dosage. Taken together, the improved egg quality may be adducible to nutrients bioavailability and efficient utilization.

### Hematological indices, antioxidant capacity, and immune function.

Hematological indices are used for health status indicators in organisms (Etim et al., 2014). Our results showed that feed additives increased RBC, WBC, Platelet and decreased H/L ratio compared to the control. The increased RBC implies that the animal is not prone to anemia and an indication of efficient nutrient utilization which in turn promotes erythropoiesis. The potentials of probiotics to cause a significant reduction in H/L was notable in the study. In a likely manner, probiotics, prebiotics or synbiotics decreased H/L ratio in laying hens (Tang et al., 2017) and broiler chickens (Salah et al., 2018). Thus, the lower H/L ratio suggests immunostimulatory nature of probiotics and the stability of animal health. On the other hand, Akinleye et al. (2008), found no effect of dietary prebiotics on hematological indices of broilers while Ghasemi et al. (2014) reported that synbiotics did not influence H/L ratio. The divergent views may be related to the hygiene condition of the farm where birds are kept. Our findings suggest that these feed additives could improve the health and welfare of the animals under farm conditions.

Serum biochemical indices are often used to measure the physiological response of the organisms to the supplemented diets. Serum immunoglobulins are used as critical indicators to assess the non-specific immunity status of the organism (Wu et al., 2019), and serum concentrations of IgA, IgM, and IgG can be modulated by dietary supplementations (Sun et al., 2020). In the current study, enhanced immunoglobulin (IgM, IgA, and IgG) concentrations due to dietary feed additives were notable. This is in line with previous evidences that dietary CB increased concentrations of IgA, IgG, and IgM (Zhan et al., 2019), IgA and IgM (Obianwuna et al., 2022b) in laying hens, and IgA, IgG, and IgM in broilers (Zhang et al., 2014). Also, FOS have been found to enhance concentrations of IgA and IgM (Ding et al., 2019), IgA (Al-Khalaifa et al., 2019) in broilers, and IgA, IgM, and IgG (Obianwuna et al., 2022a) in laying hens. In same lieu, synbiotics enhanced the concentrations of the immunoglobulins IgG in laying hens (Song et al., 2022) and IgA concentrations in laying hens challenged with salmonella (Luoma et al., 2017). The enhanced immunity status depicts the capacity of prebiotics to act as growth enhancers for probiotic gut microbes, stimulating an indirect effect on the host immune system (Xu et al., 2003). Therefore, the two-fold synergistic effect could be the immunomodulatory effect of probiotics and prebiotics. Also, we observed that the organ index of the spleen significantly improved in the probiotics group compared to the control. The result was in line with previous reports that dietary probiotics increased relative weight of the spleen in broilers (Chen et al., 2013) and spleen index in laying hens (Zhan et al., 2019). Further analysis, showed that complement proteins C3 was enhanced in the synbiotics group but no effect of dietary treatments was observable for C4. Although previous reports demonstrated that C3 and C4 concentrations were increased due to CB supplementation in laying hens (Zhan et al., 2019) and broilers (Zhang et al., 2014). The improved immune status is an indication on the immunomodulatory effects of probiotics, prebiotics and synbiotics and the potentials to support the immune system component of chickens.

Normal cellular metabolisms often lead to generation of free oxygen radicals and lipid peroxidation (Shi et al., 2020), often indicated by MDA which is an oxidative biomarker (Puvača et al., 2015). The protective effect of antioxidant enzymes (CAT, GSH-Px, GST, and T-SOD) on biological membranes from oxidative damage have been reported (Zhang et al., 2014). In the present study, supplementation of dietary probiotics, prebiotics and synbiotics significantly increased CAT, T-SOD, GSH-Px and GST, decreased MDA but no dietary treatment effect was found for T-AOC. In line with our results, literature have shown that dietary CB enhanced activities of CAT, T-SOD, GSH-Px but had no effect on T-AOC and MDA in laying hens (Zhan et al., 2019). In one study, CB reduced T-AOC but no adverse effect on the antioxidant defense system was found (Wang et al., 2021). Also, prebiotics enhanced activities of T-SOD, CAT, and GSH-Px, while decreasing MDA content (Li et al., 2017; Guo et al., 2020; Obianwuna et al., 2022a). In similar lieu, synbiotics enhanced GSH-Px, TSOD, T-AOC, reduce MDA in the serum (Song et al., 2022), decreased MDA and increased GSH but had no effect on CAT (Salah et al., 2018). The enhanced antioxidant function could probably be due to capacity of the feed additives to activate the upregulation of the enzymes in the antioxidant defense system that can scavenge ROS, and reduce lipid peroxidation (Mohammed et al., 2019). The non-significant effect of the treatments on T-AOC suggests that the treatments enhanced the antioxidant system of the host, thus lowering the activity of T-AOC. The reduced lipid peroxidation and increased enzyme activity may have reduced the susceptibility of the biological membranes to oxidative damage (Yuan et al., 2014). Therefore, this could promote the physiological response and systems crucial to egg production, egg quality and animal health. The beneficial effect of the feed additives on health of the laying hens reflects in enhanced hematological indices, immunoglobulin synthesis and antioxidant capacity, which could explain the improved laying performance and egg quality.

### Apparent fecal amino acid digestibility

There is limited information on the influence of feed additives; CB, FOS alone or in combination, on apparent fecal digestibility of crude protein and amino acids in laying hens. This study to the best of our knowledge would be the first to report the effect of dietary CB and FOS in a combined form on fecal digestibility of crude protein and amino acids in laying hens. In the present study, the fecal digestibility of crude protein and all key amino acids except proline and alanine were significantly improved in response to dietary feed additives. In the study of Emami et al. (2012), supplementation of FOS in broiler diets had no influence on crude protein digestibility while FOS enhanced methionine digestibility in cecectomized roosters (Biggs and Parsons, 2007). The variations may be due to inclusion level. Improved digestibility of crude protein and amino acids and consequent reduced loss in fecal samples, suggest that gut integrity enhanced amino acid bioavailability and absorption, rather than degradation in the gut. Prebiotics and probiotics have been found to regulate growth of beneficial microbes and suppress pathogens invasion in the gut (Kumar et al., 2019; Wang et al., 2021). It could then be that the improved microecological environment of the gut, provided support for gut integrity and nutrient absorption capacity. Amino acids including isoleucine and total sulfur amino acids (TSAA) are crucial to albumen synthesis (Phuoc et al., 2019; Parenteau et al., 2020), thus, enhanced digestibility of these amino acids may have contributed to the better albumen quality. It could be deduced that the significant improvement in crude protein digestibility, amino acid absorption and utilization, culminated in availability of circulating amino acids which the body utilized for metabolism and other physiological processes including egg production, immunoglobulins and albumen synthesis.

### Jejunal villi morphology

The small intestine is the main site for digestion and absorption of nutrients. Enhanced nutrient absorption capacity of the gut is a function of microvilli structures; shallow CD, and increased VH, VH/CD, which allows for maturity of intestinal mucosa and efficient utilization ratio of energy for gut development (Adibmoradi et al., 2006). Our findings showed that dietary probiotics, prebiotics and synbiotics, enhanced jejunal villi morphology. This corroborates the submissions of earlier reports on influence of prebiotics on jejunal villi morphology of laying hens (Ding et al., 2018; Guo et al., 2020). Also, our findings agree with previous reports in literatures that demonstrated enhancement effect of CB on ileal VH (Wang et al., 2020), VH, VH/CD and decreased CD (Xiang et al., 2019), and jejunal VH, VH/CD and decreased CD (Wang et al., 2021) in laying hens. In addition, Zarei et al. (2018) showed that the probiotic, prebiotic and synbiotics significantly increased villus height, villus height: CD ratios and decreased CD. The increased villus height, width, surface area and VH/CD, suggests an increased surface area with greater capacity for nutrient absorption (Adhikari et al., 2018). Prebiotics including FOS, acts as key substrates for probiotics in the intestinal tract, this in turn stimulates the growth of beneficial bacteria, which further promotes intestinal integrity (Sugiharto, 2016; Holscher, 2017). Probiotics (Hajiaghapour and Rezaeipour, 2018; Vieco-Saiz et al., 2019), CB (Liu et al., 2018; Wang et al., 2021), and FOS (Ding et al., 2019) are known to produce large amount of SCFAs, which provide energy for the intestinal epithelial cells (Zou et al., 2019; Salvi and Cowles, 2021), and prevention of pathogen adhesion to the GIT, thus increasing synbiotic intestinal microflora that supports villi development. Therefore, the enhanced gut morphology and two-fold synergistic effect of the synbiotics is attributable to adequate nutrient supply and beneficial microecological environment. However, Samli et al. (2007) found no significant effect of synbiotics (whey powder plus probiotics), on gut morphology of broilers. Also, FOS had no significant effect on the ileal morphology of laying hens (Adhikari et al., 2018). The variations could be due to microbial composition of the feed supplements. It could be deduced that the improved gut function increased bioavailability of nutrients, which reflect in enhanced crude protein and amino acid digestibility, egg production rate, albumen synthesis, and health status of the birds.

## Conclusion

Dietary supplementation of probiotics; CB, prebiotics; FOS on a singly basis and a combination of FOS and CB as synbiotics enhanced laying performance, albumen quality, amino acid digestibility, jejunal villi morphology, immune response and antioxidant capacity in laying hens at peak phase. The significant effect of synbiotics on albumen quality and physiological response of the birds suggests the superiority of the synbiotics over singly basis of probiotics or prebiotics. The study shows that CB and FOS either singly or combined could be used as one of dietary strategies to enhance physiological health, egg production, and albumen quality of laying hens, thus boosting poultry production.

## Data availability statement

The original contributions presented in the study are included in the article, further inquiries can be directed to the corresponding authors.

## Ethics statement

The animal study was reviewed and approved by Animal Care and Use Committee of the Feed Research Institute of the Chinese Academy of Agricultural Sciences, Beijing, China (CAAS No. 20200507).

## Author contributions

KQ, LH, and SW: Conceptualization. KQ, LH, and UO: Resources data. UO: Writing original draft. KQ, JW, HZ, LH, and SW: Supervision. KQ and UO: Writing and Editing. SW and GQ: Funding. This final version of the manuscript has been reviewed and accepted by all the authors.

## Funding

This study was supported by Beijing Innovation Consortium of Agriculture Research System, the National Key Research and Development Program of China (2022YFC2105005), the National Natural Science Foundation of China (32072774), and the Agricultural Science and Technology Innovation Program (CAAS-ZDRW202111).

## Conflict of interest

L-lH was employed by Wilmar (Shanghai) Biotechnology Research & Development Centre Co., Ltd., Shanghai, China.

The remaining authors declare that the research was conducted in the absence of any commercial or financial relationships that could be construed as a potential conflict of interest.

## Publisher’s note

All claims expressed in this article are solely those of the authors and do not necessarily represent those of their affiliated organizations, or those of the publisher, the editors and the reviewers. Any product that may be evaluated in this article, or claim that may be made by its manufacturer, is not guaranteed or endorsed by the publisher.

## Abbreviations

ADFI, average feed intake
AEG, average egg weight
AH, albumen height
CB, *Clostridium butyricum*
CAT, catalase
CD, crypt depth
FCR, feed conversion ratio
FOS, fructooligosaccharide
GST, glutathione transferase
GSH-Px, glutathione peroxidase
HDP, hen day production
HU, Haugh unit
H/L, heterophil to lymphocyte ratio
IgM, immunoglobulin M
IgG, immunoglobulin G
IgA, immunoglobulin A
MCH, mean corpuscular hemoglobin
MCV, mean corpuscular volume
MDA, malondialdehyde
ROS, reactive oxygen species
RBC, red blood cells count
SCFA, short-chain fatty acid
TAO-C, total antioxidant capacity
TSOD, total superoxide dismutase
VH, Villi height
VW, villus width
VSA, villi surface area
V/C villi height to crypt depth ratio
WBC, white blood cells count
